# Tau Filaments and the Development of Positron Emission Tomography Tracers

**DOI:** 10.3389/fneur.2018.00070

**Published:** 2018-02-15

**Authors:** Michel Goedert, Yoshiki Yamaguchi, Sushil K. Mishra, Makoto Higuchi, Naruhiko Sahara

**Affiliations:** ^1^MRC Laboratory of Molecular Biology, Cambridge, United Kingdom; ^2^RIKEN Global Research Cluster, Wako, Japan; ^3^National Institute of Radiological Sciences, Chiba, Japan

**Keywords:** tau protein, Tauopathy, tau isoform, filamentous tau aggregate, cryo-electron microscopy, positron emission tomography ligand

## Abstract

A pathological pathway leading from soluble, monomeric to insoluble, filamentous Tau, is believed to underlie human Tauopathies. Cases of frontotemporal dementia are caused by dominantly inherited mutations in *MAPT*, the Tau gene. They show that dysfunction of Tau protein is sufficient to cause neurodegeneration and dementia. Extrapolation to the more common sporadic Tauopathies leads one to conclude that the pathological pathway is central to the development of all cases of disease, even if there are multiple reasons for Tau assembly. These findings are conceptually similar to those reported for beta-amyloid, alpha-synuclein and prion protein. Here, we provide an overview of Tau filaments and their positron emission tomography ligands.

## Introduction

Neurofibrillary lesions strongly correlate with cognitive deficits, making them an important therapeutic target for Alzheimer’s disease (AD) (1, 2). Dominantly inherited mutations in *MAPT*, the Tau gene, cause a form of frontotemporal dementia that can be associated with parkinsonism (FTDP-17T), showing that dysfunction of Tau protein is sufficient to cause neurodegeneration and dementia (3). In FTDP-17T, abundant filamentous Tau inclusions are present in either nerve cells or in both nerve cells and glial cells. Aβ deposits, a defining feature of AD, are not characteristic of FTDP-17T. However, there are many similarities between cases of FTDP-17T and other pure Tauopathies, such as sporadic progressive supranuclear palsy (PSP), corticobasal degeneration (CBD), argyrophilic grain disease (AGD), and Pick’s disease, especially with regard to the isoform composition of Tau filaments.

## Tau Isoforms

Tau is expressed predominantly in the central and peripheral nervous systems, where it is most abundant in nerve cell axons. It belongs to the family of Tau/MAP2/MAP4 microtubule-associated proteins. Tau is natively unfolded with a low content of secondary structure (4, 5). However, long-range contacts between N- and C-termini, as well as between both termini and the repeats (i.e., paperclip conformation), have been described (5, 6). Using single-molecule Förster resonance energy transfer, it has been shown that upon tubulin binding the repeats expand and long-range contacts between both termini and the repeats are reduced (7).

Tau can be divided into an N-terminal domain, a proline-rich region, the repeat region, and a C-terminal domain (2). The N-terminal domain projects away from microtubules (8). Residues 2–18 have been shown to be involved in a signaling cascade that inhibits axonal transport (9). The N-terminal region also binds to the C-terminus of the p150 subunit of the dynactin complex (10). The proline-rich region has seven PXXP motifs, which provide recognition sites for SH3 domain-containing proteins of the Src family of non-receptor tyrosine kinases, such as Fyn (11). Its interaction with Tau may regulate the targeting of Fyn and thereby mediate beta-amyloid-induced toxicity (12). It has been reported that the proline-rich region of Tau also mediates binding to other proteins, including bridging integrator 1 and peptidyl-prolyl *cis/trans* isomerases (13). Interactions between Tau and microtubules are mediated through the repeats and some adjoining sequences (2). Less is known about the role of the C-terminal region. Tau belongs to the family of intrinsically disordered proteins, which have many interaction partners and are commonly implicated in neurodegenerative diseases (14). Theoretical calculations have estimated that more than 70 different binding partners of tau may exist (14).

**Figure 1:**
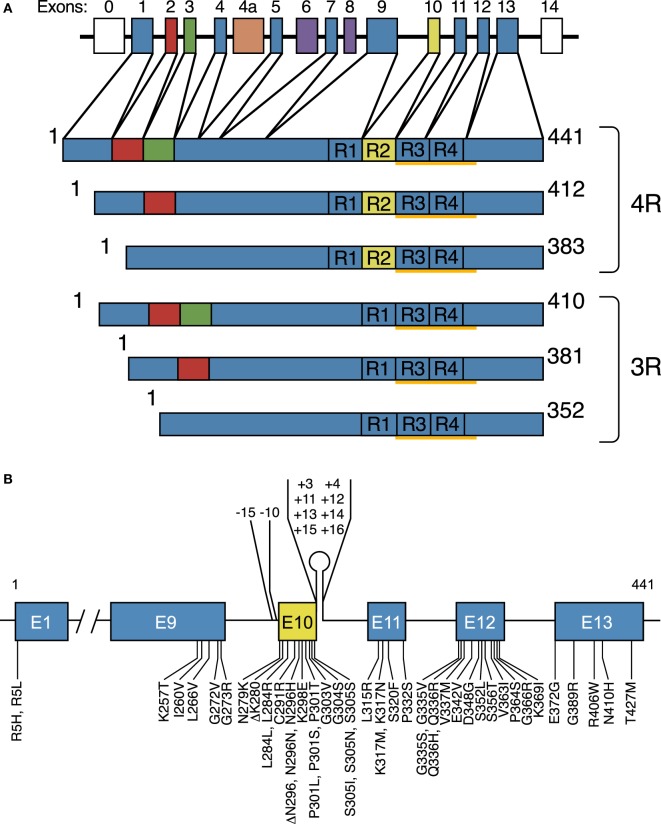
Human brain Tau isoforms and disease-causing *MAPT* mutations. **(A)** *MAPT* and the six Tau isoforms expressed in adult human brain. *MAPT* consists of 16 exons (E). Alternative mRNA splicing of E2 (red), E3 (green), and E10 (yellow) gives rise to six Tau isoforms (352–441 amino acids). The constitutively spliced exons (E1, E4, E5, E7, E9, E11, E12, and E13) are shown in blue. E0, which is part of the promoter, and E14 are non-coding (white). E6 and E8 (violet) are not transcribed in human brain. E4a (orange) is only expressed in the peripheral nervous system. The repeats (R1–R4) are shown, with three isoforms having four repeats each (4R) and three isoforms having three repeats each (3R). The core sequences of the Tau filaments from Alzheimer’s disease brain (V306-F378) determined by cryo-EM are underlined. **(B)**, Mutations in *MAPT* in cases of frontotemporal dementia and parkinsonism linked to chromosome 17 (FTDP-17T). Forty-nine coding region mutations and 10 intronic mutations flanking E10 are shown.

Six Tau isoforms ranging from 352 to 441 amino acids in length are expressed in adult human brain (Figure 1A) (15). They are produced by alternative mRNA splicing of transcripts from *MAPT* and differ by the presence or absence of inserts of 29 or 58 amino acids (encoded by exons 2 and 3 of *MAPT*, with exon 3 being only transcribed in conjunction with exon 2) in the N-terminal half, and the inclusion, or not, of the 31 amino acid microtubule-binding repeat, encoded by exon 10, in the C-terminal half. Inclusion of exon 10 results in the production of three Tau isoforms with four repeats each (4R) and its exclusion in a further three isoforms with three repeats each (3R). The repeats comprise residues 244–368 of Tau, in the numbering of the 441 amino acid isoform. The N-terminal inserts are not believed to play an active role in Tau aggregation, but the insert encoded by exon 10 is important. In adult human brain, similar levels of 3R and 4R Tau are expressed (16), and the finding that a correct isoform ratio is essential for preventing neurodegeneration (17, 18) came as a surprise. Inclusion of exons 2 and 3, giving rise to 2N isoforms, is relatively underrepresented in comparison with inclusion of exon 2 and exclusion of exons 2 and 3, such that 2N, 1N, and 0N Tau isoforms make up 9, 54, and 37% of the total.

Why six Tau isoforms are found in adult human brain is not known. Isoform expression is not conserved between species (19–22). Thus, in adult mouse brains, 4R Tau isoforms are almost exclusively present, whereas adult chicken brains express 3R, 4R, and 5R Tau isoforms. However, what is conserved is the expression of one hyperphosphorylated 3R Tau isoform lacking N-terminal repeats during vertebrate development. In mice the switch from 3R to 4R Tau occurs between postnatal days 9 and 18, with Tau phosphorylation also decreasing during that time (23). However, isoform switching and phosphorylation are regulated differently. Adult Tau isoforms with 4R are better at promoting microtubule assembly and binding to microtubules than the fetal 3R Tau isoform (16). This is consistent with the need for a more dynamic cytoskeleton during the development of nerve cells.

The repeats and some adjoining sequences constitute the microtubule-binding domains of Tau. Single-molecule tracking revealed a kiss-and-hop mechanism, with a dwell time of Tau on individual microtubules of only about 40 ms (24, 25). Isoform differences did not influence this interaction. Despite these rapid dynamics, Tau promoted microtubule assembly. It remains to be seen if microtubules were also stabilized. In brain, Tau is subject to a large number of posttranslational modifications, including phosphorylation, acetylation, methylation, glycation, isomerization, *O*-GlcNAcylation, nitration, sumoylation, ubiquitination, and truncation (26–28). Big Tau, which carries an additional large exon in the N-terminal half, is only expressed in the peripheral nervous system (29, 30). Several structural models have been put forward for the binding of Tau to microtubules (31–33), but there is no consensus. Overall, it appears that the microtubule-bound conformation of Tau may delay aggregation. Cryogenic electron microscopy (cryo-EM) is bound to provide atomic structures of Tau bound to microtubules that were assembled from tubulin in different ways (34).

## Tau Filaments

Full-length Tau assembles into filaments through its repeats, with the N-terminal half and the C-terminus forming the fuzzy coat (35–38). Tau filaments from human brain and those assembled from expressed protein have a cross-β structure characteristic of amyloid fibrils, with their cores consisting of approximately 90 amino acids (39). The region of Tau that binds to microtubules also forms the core of Tau filaments, suggesting that physiological function and pathological assembly are mutually exclusive.

Phosphorylation of Tau negatively regulates its ability to interact with microtubules, and filamentous Tau is abnormally hyperphosphorylated (40). However, it remains to be proved that phosphorylation is the trigger for aggregation in human diseases. Alternatively, a conformational change in Tau arising from assembly may cause its hyperphosphorylation. Recombinant Tau assembles in bulk into filaments when incubated with heparin, in the absence of phosphorylation (41, 42). However, it has also been shown that recombinant S262A 4R Tau assembled into filaments following incubation with brain extracts from adult rats (43). Other posttranslational modifications may also be involved. Initial studies on Tau acetylation reported that it promoted phosphorylation and aggregation (44, 45). However, subsequent work has suggested that an inverse correlation exists between Tau acetylation and phosphorylation, with acetylation inhibiting Tau assembly (46, 47). Unlike phosphorylation, acetylation occurs on lysine residues, as do glycation, ubiquitination, and methylation.

Many publications equate Tau phosphorylation with aggregation. This is probably not correct. Although aggregated Tau is heavily phosphorylated in human brain, not all phosphorylated Tau is aggregated or on its way to aggregation. For instance, highly phosphorylated Tau forms during hibernation, in the absence of aggregation (48). There is substantial overlap between the phosphorylation of Tau during development and its hyperphosphorylation in disease. However, some Tau phosphorylation, such as that at T212, S214, and T217 detected by antibody AT100, is pathological (49). Antibody AT8 has been used to detect both physiologically and pathologically phosphorylated Tau. It was recently shown that it recognizes triply phosphorylated Tau (S202, T205, and S208) better than doubly phosphorylated protein (S202 andT205), raising the possibility of differential phosphorylation of pathologically and physiologically phosphorylated Tau at the AT8 epitope (50).

In AD, chronic traumatic encephalopathy, postencephalitic parkinsonism, and many other Tauopathies, all six isoforms are present in the disease filaments (Table 1) (2). They are either paired helical (PHFs) or straight (SFs) and contain both 3R and 4R Tau isoforms in a one-to-one ratio, similar to the isoform composition and relative abundance of the six isoforms in soluble Tau from normal human brain. By cryo-EM, the cores of Tau filaments from AD are made of two identical protofilaments consisting of residues V306-F378 of Tau, which adopt a combined cross-β/β-helix structure, possibly defining the seed for Tau aggregation (51). The N-terminal part of the cross-β structure is formed by the hexapeptide ^306^VQIVYK^311^ (PHF6), which is essential for the oligomerization of Tau and its assembly into filaments (52, 53). It packs through a heterotypic, non-staggered interface with the opposing residues 373–378. The same packing interface is absent in the widely used K18 and K19 proteins, which span three or four repeat domains of recombinantly expressed Tau, and end at E372 (54). Therefore, filaments made of K18 and K19 proteins cannot represent the complete core structure of PHFs and SFs from the brains of individuals with AD. The second hexapeptide motif ^275^VQIINK^280^ (PHF6*) that is required for filament assembly (55) does not form part of the core of Tau filaments from AD brain. However, inhibitors of the PHF6* motif have been shown to reduce the heparin-induced assembly of 4R Tau (56). Both hexapeptide motifs were required for the seeded aggregation of mutant human Tau in transfected non-neuronal cells (57). It remains to be seen if PHF6 and PHF6* are required for the assembly of Tau in human brain.

**Table 1:** Neurodegenerative diseases with abundant tau inclusions.

**3R + 4R Tauopathies**
Alzheimer’s disease
Amyotrophic lateral sclerosis/parkinsonism-dementia complex
Anti-IgLON5-related Tauopathy
Chronic traumatic encephalopathy
Diffuse neurofibrillary tangles with calcification
Down’s syndrome
Familial British dementia
Familial Danish dementia
Gerstmann–Sträussler–Scheinker disease
Niemann–Pick disease, type C
Non-Guamanian motor neuron disease with neurofibrillary tangles
Postencephalitic parkinsonism
Progressive ataxia and palatal tremor
Tangle-only dementia
Familial frontotemporal dementia and parkinsonism (some *MAPT* mutations, such as V337M and R406W)
**3R Tauopathies**
Pick’s disease
Familial frontotemporal dementia and parkinsonism (some *MAPT* mutations, such as G272V and Q336R)
**4R Tauopathies**
Argyrophilic grain disease
Corticobasal degeneration
Guadeloupean parkinsonism
Globular glial Tauopathy
Huntington’s disease
Progressive supranuclear palsy
SLC9A6-related parkinsonism
Tau astrogliopathy
Familial frontotemporal dementia and parkinsonism (some *MAPT* mutations, such as P301L and P301S, all known intronic mutations, and many coding region mutations in exon 10)

Each protofilament contains eight β-strands, five of which give rise to two pairs of anti-parallel β-sheets, with the other three forming a β-helix. PHFs and SFs differ in their inter-protofilament packing, showing that they are ultrastructural polymorphs. The protofilaments of PHFs are arranged base-to-base, whereas those of SFs are arranged back-to-base. These findings do not explain why all six Tau isoforms are found in PHFs and SFs. However, a less ordered β-sheet is present upstream of V306; it can accommodate an additional 16 amino acids, which probably correspond to a mixture of residues 259–274 (R1) from 3R Tau and 290–305 (R2) from 4R Tau.

In other diseases, such as PSP, CBD, AGD, globular glial Tauopathy, and aging-related Tau astrogliopathy, isoforms with 4R Tau are found in the filaments (Table 1) (3), but the presence of 3R Tau-positive neuronal inclusions has also been reported in PSP and CBD (58, 59). The Pick bodies of Pick’s disease are only made of 3R Tau (Table 1) (60). The morphologies of Tau filaments in different diseases vary, even when they are made of the same isoforms. Silver staining can also detect these differences (61). Inclusions made of all six Tau isoforms stain with Gallyas–Braak and Campbell–Switzer. Those made of 4R Tau are only positive with Gallyas–Braak, whereas those made of 3R Tau stain only with Campbell–Switzer. It remains to be seen if the cores of filaments made of 3R or 4R Tau differ structurally from those of AD, which are made of 3R + 4R Tau isoforms.

The specificity of antibodies Alz50 and MC-1 for assembled Tau relies on a conformation that all isoforms can undergo and which requires two discontinuous intramolecular epitopes separated by almost 300 amino acids (62, 63). They are ^7^EFE^9^ in the N-terminus and ^313^VDLSKVTSKC^322^ in R3. MC-1 staining is one of the earliest markers of misfolded Tau. NMR experiments using heparin-induced filaments of 4R Tau also provided evidence for an interaction between the N-terminus and residues 313–322 of the structured core (64). Moreover, the cryo-EM structures of Tau filaments from AD brain showed a density consistent with ^7^EFE^9^ contacting K317 and K321 in the protofilament core (51). These electrostatic interactions may be essential for Tau filament formation, implying that acetylation of K317 and/or K321 might protect against aggregation. The only known disease-causing mutations in *MAPT* that are located outside the repeats and the C-terminus (R5H and R5L) (3) are close to ^7^EFE^9^.

**Figure 2:**
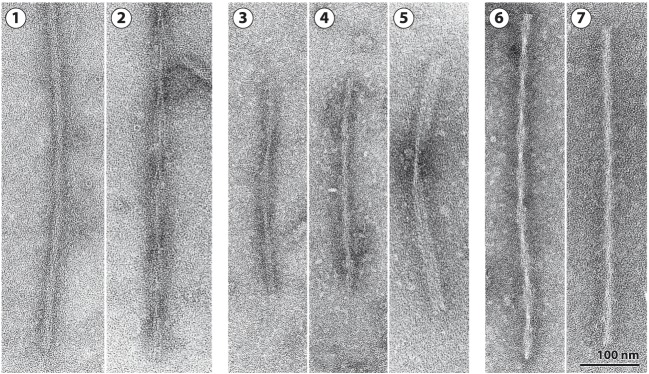
Tau filaments from FTDP-17T. [1,2] Neuronal Tau filaments from a case with abundant Pick body-like inclusions and a G389R mutation in *MAPT* (66). [1] Straight filaments form the majority species and [2] strongly stranded twisted filaments are in the minority. [3–5] Tau filaments from cases with neuronal and glial inclusions and a P301L mutation in *MAPT* or an intronic mutation [from Ref. (67, 68)]. [3] Narrow twisted ribbons and [4] occasional rope-like filaments. [5] Familial multiple system Tauopathy with presenile dementia and other cases caused by *MAPT* mutations in the intron after E10 are characterized by wide twisted ribbons and neuronal and glial Tau inclusions. The filaments in [3–5] are made of 4R Tau. [6,7] Tau filaments from a case with a V337M mutation in *MAPT* [from Ref. (69)]. [6] Paired helical and [7] straight filaments are present as in Alzheimer’s disease. Tau inclusions are largely neuronal, and filaments in [6] and [7] are made of 3R and 4R Tau.

Fifty-nine different mutations in *MAPT* have been identified in FTDP-17T (Figure 1B) (3). The filaments consist of 3R, 4R, or 3R + 4R Tau (Figure 2) (65). *MAPT* mutations account for approximately 5% of cases of frontotemporal dementia and are concentrated in exons 9–12 (encoding R1–R4) and the introns flanking exon 10. They can be divided into those with a primary effect at the protein level and those affecting the alternative splicing of Tau pre-mRNA. There is no obvious correlation between known mutations and posttranslational modifications of Tau.

It has been suggested that patients with AD-type neurofibrillary degeneration restricted to hippocampus and medial temporal lobe, who lack Aβ deposits, suffer from primary age-related Tauopathy (PART), a condition that differs from AD (70). Tangle-only dementia, a rare form of dementia, may represent a severe form of PART (71). However, the view that PART is different from AD has been challenged, because it is clinically and neuropathologically similar to what appear to be the early stages of the Tau pathology of AD (72).

In AD, following the death of tangle-bearing cells, Tau filaments can remain in the extracellular space as ghost tangles, which consist largely of Tau repeats that have lost their fuzzy coat through proteolysis. In Pick’s disease, PSP, CBD, and most cases caused by *MAPT* mutations, Tau filaments do not accumulate to a significant extent in the extracellular space following the death of aggregate-bearing cells. The reasons why Tau filaments from AD brain are less soluble remain to be established (2).

## Tau Aggregate-Binding Ligands and the Development of Positron Emission Tomography (PET) Tracers

Monomeric Tau assembles into filaments through oligomerization (1, 2). In tissue sections, filamentous Tau aggregates are labeled by amyloid-binding dyes, such as Congo red, thioflavins, and some luminescent conjugated oligothiophenes (2, 73). These dyes appear to bind to both intra- and extracellular Tau deposits. They are useful for cross-sectional studies but require the availability of brain tissue.

To perform longitudinal studies and to assess the effects of treatments on the level of aggregates, one needs to be able to visualize Tau inclusions repeatedly in the living human nervous system. The field of PET imaging of brain inclusions characteristic of human neurodegenerative diseases started with the development of [^11^C]Pittsburgh compound B ([^11^C]PIB), a derivative of thioflavin T, which detects β-amyloid deposits in the living brain (74). Subsequently, several PET tracers for aggregated Tau, such as [^11^C]PBB3, [^18^F]PM-PBB3, [^18^F]AV-1451, [^18^F]THK5351, [^18^F]MK-6240, [^18^F]R06958948, [^18^F]GTP-1, and [^18^F]PI-2620, were developed and are currently being tested in humans (75–81). Most tracers show a high affinity for Tau inclusions and recognize β-amyloid deposits less well (79, 81). However, some off-target effects have also been described. Thus, non-specific retention of [^11^C]PBB3 was seen in the dural venous sinuses (75). *In vitro* studies have shown that [^18^F]AV-1451 can bind to monoamine oxidase (MAO)-A, as well as to pigmented and mineralized vascular structures (82). Retention of [^18^F]AV-1451 in the choroid plexus of control individuals also reflected off-target binding (83). Age-related, off-target effects of [^18^F]AV-1451 binding in the basal ganglia closely correlated with iron accumulation (84). Selegiline, a MAO-B inhibitor, reduced [^18^F]THK5351 signal in basal ganglia and neocortex (85). Moreover, an *in vitro* study confirmed that MAO-B was an off-target binding substrate for [^18^F]THK5351 (86). Perhaps most worryingly, elevated binding of [^18^F]AV-1451 and [^18^F]THK5351 has been described in the semantic variant of primary progressive aphasia, a form of frontotemporal dementia that is consistently associated with assembled TDP-43, but not with Tau inclusions (87–89). Where studied, second generation Tau PET tracers ([^18^F]PM-PBB3, [^18^F]MK-6240, [^18^F]R06958948, [^18^F]GTP-1, and [^18^F]Pl-2620) have shown less off-target binding than the first generation of tracers. Future autopsy studies are needed to identify the binding targets of these ligands. On the other hand, the distribution of [^18^F]AV-1451 binding, a first generation tracer, recapitulated Braak staging in AD brain (90). Moreover, a combination of PET imaging with [^18^F]AV-1451 and graph theory supported the view that tau pathology can undergo transneuronal spread (91), consistent with experimental studies (92, 93).

**Figure 3:**
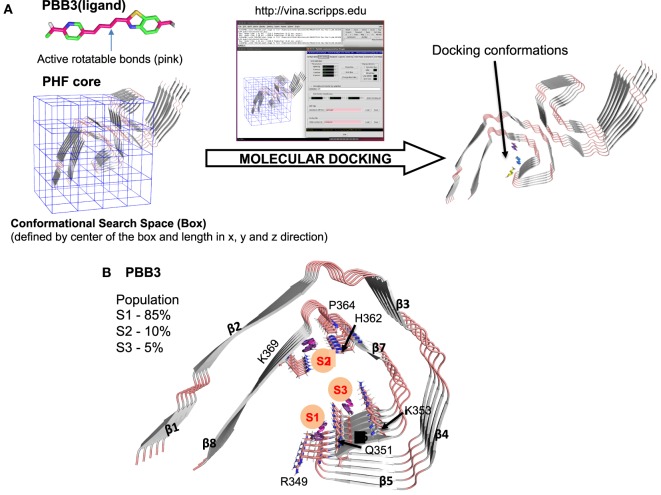
Molecular docking of PBB3 to paired helical Tau filaments (PHFs) from Alzheimer’s disease brain. **(A)** Schematic representation of the docking process using Pymol and AutoDock Vina. The PHF core structure was from Ref. (51) (PDB ID: 503L). Out of 100 docking conformations, the top 20 were selected for further analysis. **(B)** Molecular docking of PBB3 into the PHF protofilament core structure. The top 20 conformations distributed into three clusters (S1, S2, and S3). S1 had the highest affinity for the Tau filament, followed by S2 and S3.

To develop more specific and selective ligands, it is important to determine where in the structured cores of Tau filaments PET ligands bind. Recent advances in cryo-EM, which have resulted in the determination of the high-resolution structures of Tau filaments from AD brain (51), have made this possible in principle. We used this information, together with molecular docking (94), to study the binding of PBB3 to the protofilament core of Tau filaments from AD (Figure 3A). As shown in Figure 3, PBB3 bound in a perpendicular manner to a high-affinity site (S1) in the C-shaped part of the protofilament, which includes residues 349–351 (RVQ) of Tau (Figure 3B). Two lower affinity binding sites were also detected, at residues 364–369 (PGGGNK) (S2) and 351–353 (QSK) (S3) (Figure 3B).

PBB3 visualizes the Tau pathologies of AD and non-AD Tauopathies (75, 95, 96). Unlike PBB3, previous *in vitro* and *in vivo* studies have shown that AV-1451 binds only with low-affinity to filaments from non-AD Tauopathies (95, 97, 98). It has been reported that AV-1451 and its lead compound failed to visualize Tau inclusions in a mouse line transgenic for human P301L Tau (76). However, using [^11^C]PBB3, it was possible to image Tau inclusions in mouse models of Tauopathy (lines PS19 and Tg4510) (75, 99). These findings further support the view that AV-1451 recognizes Tau inclusions made of 3R or 4R Tau with lower affinity than those made of 3R + 4R Tau. It will be interesting to obtain cryo-EM structures of the cores of Tau filaments from AD and other Tauopathies with bound PET ligands. One cannot exclude that high-affinity binding sites exist in the “fuzzy coat” of human brain Tau filaments. However, both AV-1451 and PBB3 have been shown to detect extracellular Tau inclusions in AD brain (95). We believe that the aggregated Tau in extracellular tangles corresponds closely to the structured filament cores.

## Conclusion

The determination of high-resolution structures of Tau filaments by cryo-EM has opened the way for elucidating the structures of other amyloid filaments from human brain. Future work will tell what the differences between morphotypes of amyloid filaments are, which will in turn inform the mechanisms underlying the prion-like propagation of protein aggregates. Perhaps most importantly, cryo-EM will make it possible to relate mechanisms of amyloid formation of recombinant proteins to those in human brain.

## Author Contributions

MG and NS wrote the manuscript; YY and SM analyzed the molecular docking simulation; and MH critiqued the manuscript.

## Conflict of Interest Statement

The authors declare that the research was conducted in the absence of any commercial or financial relationships that could be construed as a potential conflict of interest.
